# Sodium butyrate protect bone mass in lipopolysaccharide-treated rats by reducing oxidative stress and inflammatory

**DOI:** 10.1080/13510002.2024.2398891

**Published:** 2024-09-16

**Authors:** Zhou-Shan Tao, Tao Ma

**Affiliations:** a Department of Orthopedics, The First Affiliated Hospital of Wannan Medical College, Yijishan Hospital, Wuhu, People’s Republic of China; b Anhui Province Key Laboratory of Non-coding RNA Basic and Clinical Transformation, Wuhu, People’s Republic of China

**Keywords:** Lipopolysaccharide, oxidative stress, sodium butyrate, inflammation, bone mass, bone metabolism, bone formation, bone resorption‌

## Abstract

**Objective:**

The study will be to observe the effect of Sodium butyrate (NaB) on bone loss in lipopolysaccharide (LPS)-treated rats.

**Methods:**

In the rat model, we observed that changes in the expression of oxidative stress regulators, inflammatory markers and target genes were measured by immunofluorescence and RT–PCR after treatment. Changes in viability and osteogenesis of MC3T3-E1, osteoclast differentiation in RAW264.7 cells in the presence of LPS were evaluated using CCK-8, ALP staining, RES staining, and TRAP staining.

**Results:**

In vitro experiments have shown that LPS-induced inhibition of JC-1, SIRT1, GPX1 and SOD2 is associated with increased levels of inflammation and oxidative stress. In addition, NaB has been found to suppress oxidative stress, inflammation and Mito SOX, promote osteogenic differentiation, and inhibit osteoclast differentiation. In addition, NaB significantly promoted SITR1 expression, repaired impaired bone metabolism, and improved bone strength and bone mineral density.

**Conclusion:**

Given all this experimental evidence, the results strongly suggest that NaB can restore osteogenic activity in the presence of LPS by reducing intracellular ROS, inhibiting osteoclast differentiation and reducing bone loss in LPS-treated rat models.

## 1. Introduction

Osteoporosis, a systemic metabolic disease of the bones, is caused by a number of factors and typically manifests itself as decreased bone mass and increased bone frailty [1]. As the population ages, the prevalence of osteoporosis and incidence of fracture are increasing, bringing significant morbidity, loss of independence, and death [2]. Therefore, in an aging population, osteoporosis has become a major public health problem [3,4]. There have been numerous dramatic advances in osteoporosis prevention and treatment over the past decade, particularly in drug therapy. Nevertheless, osteoporotic fracture closely related to the high disability rates in elderly patients, even with the development of technology for internal fixation and surgical technology solutions [5]. The development of osteoporosis results in reduced ability to self-repair, reduced fracture union, poor osseointegration, and progressive loosening of joints after joint replacement [6,7]. Osteoporosis has become a major global public health problem as life expectancy and the proportion of older people increase, and how to reduce the risk of osteoporosis fractures presents challenges for patients and physicians [7].

In the pathological mechanism of bone mass loss induced by lipopolysaccharide (LPS), many works suggest that inflammation and oxidative stress are believed to play a key role in this process [8,9]. Studies have shown a strong correlation exists between oxidative stress and severity of osteoporosis, which indicate that imbalance of bone metabolism may substantially contribute to bone loss under inflammation and oxidative stress elicited by LPS [10,11]. Moreover, LPS induces inflammation in osteoblasts, which not only stimulates apoptosis, but also triggers loss of osteogenic differentiation [12]. To reduce bone loss in osteoporotic bones induced by LPS, pharmacological interventions have been individually investigated. Sodium butyrate (NaB), first considered to be produced by fermentation from the gut microbiota of the large intestine [13], exerts multiple protective effects by improving specific antioxidant enzymes [14]. Previous studies have shown that NaB can effectively minimize mitochondrial dysfunction through mitochondrial oxidative phosphorylation [15,16]. In addition, NaB has been found to promote bone formation, stimulate bone tissue metabolism, increase bone mineral density (BMD), and inhibit bone loss [17]. Due to its antioxidant and anti-inflammatory effect, drugs can with resistance to oxidative stress can inhibit bone mass loss and improve bone strength in ovariectomized rats [18,19]. However, whether NaB can resist the adverse effects of LPS on bone loss remains ambiguous.

Numerous studies have confirmed the therapeutic potential and molecular mechanisms of NaB in oxidative stress. However, it is beyond our knowledge whether NaB has positive effects on bone remodeling in LPS-induced osteoporosis through inhibiting inflammation and oxidative stress. ⁣Therefore, based on a rat model of LPS-induced osteoporosis, we wanted to explore the effects of NaB on bone health.

## 2. Materials and methods

### 2.1. Animal experiment

3-month-old male Sprague–Dawley(SD) rats(weighing 230–260 g) were kept in stainless steel cages and be free to tab water and standard rodent chow under pathogen-free conditions and a 12-h light – dark cycle at the constant temperature of 23 °C. These experimental animals were randomly assigned to three groups (with ten rats in each group): Control group (Con), lipopolysaccharide treatment group (Sigma-Aldrich, LPS) and LPS plus sodium butyrate (Sigma-Aldrich) treatment group (NaB + LPS). The rats from LPS and NaB + LPS were treated with NaB at a dose of 4% sodium butyrate mg/kg/day (Sigma-Aldrich) and were intraperitoneally injected with 5 mg/kg/day LPS (Sigma-Aldrich, USA) according to previous reports [20,21]. At 12 weeks of treatment, all rats were sacrificed and their blood and bones were preserved for subsequent evaluation. All experiments were approved and conducted in strict compliance with international standards regarding animal welfare, under the supervision of the Institutional Animal Care Committee at Wannan Medical College (Approval No. LLSC-2020-082).

### 2.2. Micro-CT evaluation

The right distal femur were scanned by Bruker Skyscan micro – computed tomography (CT) system (Skyscan 1176, Kontich, Belgium) as previously described [22,23]. The scanning parameters were set as follows: voltage, 70 kV; electric current, 114 μA; and resolution 10 μm. Trabecular bone microarchitecture were evaluated starting approximately 2.0 mm proximal from the femoral condyles and obtained 100 slices (1.0 mm total), producing images for analysis. After 3D reconstruction, bone mineral density(BMD), bone volume fraction(BV/TV), trabecular number(Tb.N), trabecular thickness(Tb.Th), trabecular separation(Tb.Sp), the mean connective density (Conn.D) were used to assess trabecula bone changes of distal femur.

### 2.3. Mechanical test

The femurs(N = 5) undergo a three-point bending test using a universal testing system (Instron 3360, Instron Co., High Wycombe, UK). The left femurs are first fixed to two support points and keep the indenter in the middle. The specimens were then subjected to a three-point bending test, with the distance between the two supports set at 20.0 mm. All mechanical properties including the energy to failure, maximum load, and stiffness were calculated from the load-displacement curves according to previous report [24].

### 2.4. Histological evaluation

Following micro-CT scanning, the femur specimens were decalcified in 10% ethylene diaminetetraacetic acid (EDTA) for 4 weeks and stained with HE and Masson to visualize the changes of trabecular structure of distal femur according to previous reports [22,23]. Double immunofluorescence staining for SIRT1 and SOD2 was performed to detect the levels of oxidative stress in bone tissue. After blocking with 5% (w/v) BSA for 1 h, the paraffin bone sections were incubated with anti – SIRT1 (1:100; Abcam, UK) and anti – SOD2 (1:100; Abcam, UK) overnight at 4°C, and then incubated with fluorophore-conjugated secondary antibodies (1:200, Abcam, UK) for 1 h at 25°C. Cell nuclei were stained with DAPI. For observation and image acquisition a fluorescence microscope was used, and the mean intensity of optical density was calculated semiquantitatively using Image Pro Plus software.

### 2.5. Measurement of bone metabolism index, oxidative stress index and inflammatory markers

The serum levels of osteocalcin (OC) and tartrate-resistant acid phosphatase (TRAP), Tumor necrosis factor-α (TNF-α), and interleukin-1β (IL-1β), superoxide dismutase 2 (SOD 2), malondialdehyde (MDA), total antioxidant capacity (TAC) were evaluated with commercial ELISA kits (Nanjing Jiancheng Bioengineering Institute) according to the manufacturers instructions.

### 2.6. MC3T3-E1 cell and raw264.7 cell experiments

In this study, RAW264.7 cell and MC3T3-E1 was used for the in vitro, which was purchased from Shanghai Cell Bank. Cells were seeded at normal medium (DMEM) with a density of 1 × 10^4^/ml and cultured with different environment as follows: control group (Con); LPS group at 2 μg/mL treatment (LPS); LPS plus NaB (Sigma-Aldrich, MO) at 0.3 mM treatment (NaB + DM; Sigma); diabetic milieu plus 0.3 mM NaB and EX527 (a SIRT1 inhibitor, 10 μM; Selleck Chemicals, Houston, TX, USA) treatment group (NaB + DM + EX527). Dosages used for LPS, EX527 and NaB were chosen according to previous studies [20,25,26].

The Cell Counting Kit 8 (CCK8, Dojindo, Japan) was used to assess MC3T3-E1 cell proliferation as described in previous reports [20,25,26]. Briefly, 1x 10^4^ MC3T3-E1 was seeded into each well of a 24-well plate and treated with NaB for 72 h. Next, 10% CCK-8 solution was added to the culture medium for 4 h in 37°C and examined the absorbance values by using a spectrophotometer (BioTek, Winooski, VT).

Afterwards, the osteogenic direction of the MC3T3-E1 was induced by osteogenic differentiation medium (0.1 µM dexamethasone, 50 µM ascorbate-2 phosphate, 10 mM glycerophosphate (Sigma)). MC3T3-E1 was seeded onto 6-well plates with 1 × 10 ^5^ cells /well. When the cells reached ∼70–80% confluence, osteogenic differentiation media and culture was carried out for 14 or 21 days. Following this, the treated cells were analyzed with alkaline phosphatase (ALP) staining and alizarin red (AR) staining to determine osteoblast differentiation.

Next, RAW264.7 cells were induced to differentiate into osteoclasts. RAW264.7 was seeded onto 6-well plates with 1 × 10^5^ cells /well. When the cells reached ∼70–80% confluence, 100 ng/ml of RANKL was added to media and culture was carried out as described previously [27]. Then, different intervention factors were added to induce RAW264.7 cells to differentiate into osteoclasts for 7 days, and TRAP staining (Sigma) was performed to evaluate the differentiation process.

### 2.7. Evaluation of oxidative stress and mitochondrial membrane potential(JC-1)

The changes of oxidative stress were stained with 2’,7’-dichlorofluorescein diacetate (DCFH-DA, Sigma, St. Louis, MO) and MitoSOX™ Red mitochondrial superoxide indicator(MitoSOX™, Sigma, St. Louis, MO) according to the manufacturer’s instructions. SIRT1 staining(1:100; Abcam, UK) and SOD2 staining(1:100; Abcam, UK) were performed to evaluate the density of osteoclasts in the callus area following a standard protocol. At the same time, a concentration of 30μM H_2_H_2_ hydrogen peroxide was utilized as the positive control group. Measurement processes were performed as above described [28,29].

In the fluorescence-based assay, red fluorescence indicates a normal mitochondrial membrane potential, while an abnormal mitochondrial membrane potential exhibits green fluorescence and indicates mitochondrial dysfunction and apoptosis [30]. After 48 h of different interventions, the change in ΔΨm was detected using a MitoProbe JC-1 Assay Kit (Invitrogen) according to the manufacturer’s instructions. Mitochondrial membrane potential was analyzed using 10 nm MitoTracker Green (Life Technologies) and 20 nm tetramethylrhodamine methyl ester (TMRM; Life Technologies).

### 2.8. Reverse Transcription-Polymerase Chain Reaction (RT–PCR)

Total RNA of osteoblasts after different therapeutic interventions was extracted using TRIzol reagent (Invitrogen), and a commercial PrimeScript reverse transcription kit (TaKaRa, Tokyo, Japan) was purchased for cDNA preparation. Subsequently, Real-time PCR amplification reactions were carried out through the SYBR Green PCR Kit (TaKaRa). Relative expression of target gene (Table 1) was determined by calculating Ct (thresh-old cycle) values and normalized by glyceraldehyde-3-phosphate dehydrogenase (GAPDH) levels.

**Table 1.** The primer sets used in this research are collected in Table 1 .

Gene	Accession number in Gene Bank	Forward Primer sequence(5′−3′)	Reverse Primer sequence(5′−3′)
IL-1β	**NC_000068.8**	TCATTGTGGCTGTGGAGAAGC	AATGGGAACGTCACACACCAG
SOD1	**NT_037436.4**	GGTGGGCCAAAGGATGAAGAG	CCACAAGCCAAACGACTTCC
SOD2	**NC_000006.12**	GGGGATTGATGTGTGGGAGCACG	AGACAGGACGTTATCTTGCTGGGA
CAT	**NC_058377.1**	TGGGATCTCGTTGGAAATAACAC	TCAGGACGTAGGCTCCAGAAG
TNF-α	**NC_000083.7**	TCCCCAAAGGGATGAGAAGTT	GAGGAGGTTGACTTTCTCCTGG
SIRT1	**NC_000076.7**	GCGGGAATCCAAAGGATAAT	CTGTTGCAAAGGAACCATGA
GAPDH	**NC_000012.12**	AGAAAAACCTGCCAAATATGATGA	CTGGGTGTCGCTGTTGAAGTC

### 2.9. Statistical analysis

All data were presented as the means and standard deviations using the SPSS 21.0 software. The data were assessed for normality in order to determine the appropriate use of either parametric or non-parametric tests. All parametric data used in this study were analyzed One-way analysis of variance (One way ANOVA) was used for multiple group comparisons followed by Tukey’s post hoc test and expressed as the means ± SD with n ≥ 3. Significance was measured at the following thresholds: *P < 0.05.

## 3. Results

### 3.1. NaB prevents bone loss in the distal femur in LPS-treated rats

**Figure 1.**
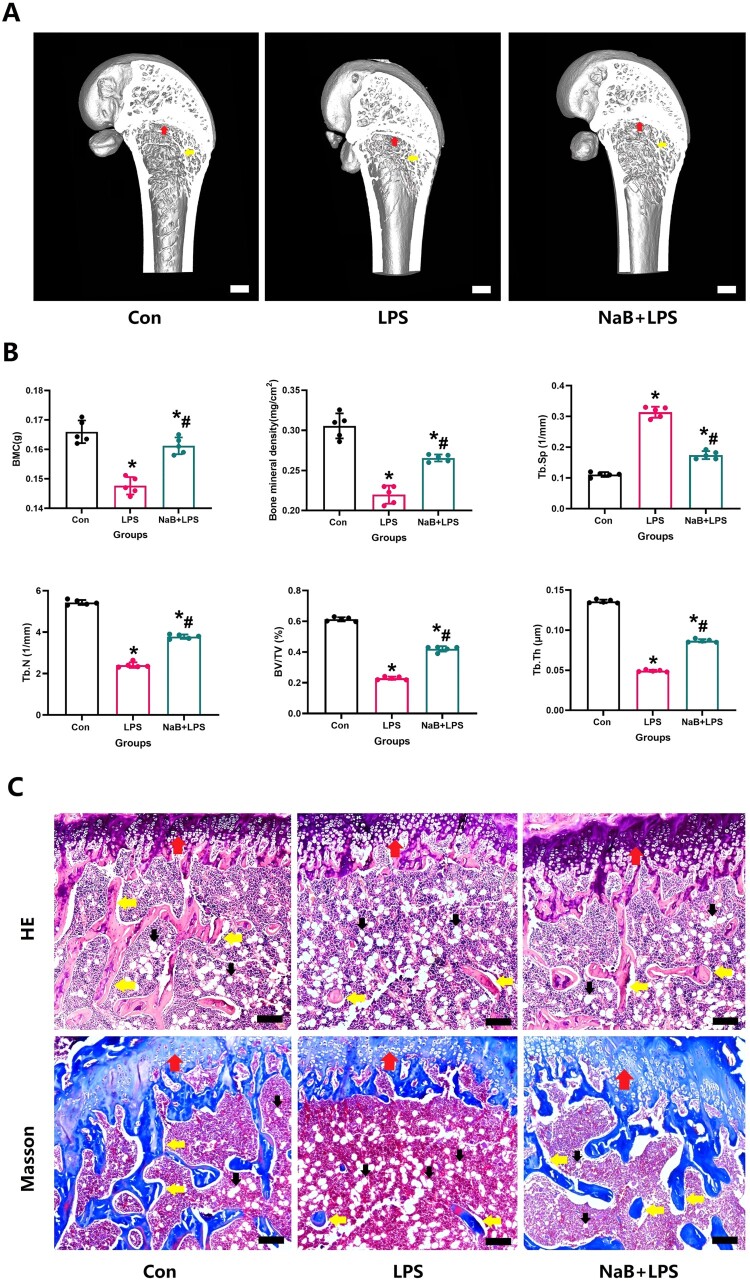
Results from micro-CT and 3D reconstruction, HE and Masson stainings were used to illustrate that NaB therapy significantly reduced bone loss in LPS-treated rats (scale bar = 1 mm). A: Representative Micro-CT 3D reconstruction and scan images after 12 weeks treatment from groups Con (a), LPS (b) and NaB + LPS (c). B: The quantitative parameters including BMD, BV/TV, Tb. Th, Tb. N, Conn. D and Tb. Sp(N = 5). C: Systemic administration with NaB could reduce bone loss in LPS-treated rats with using HE and Masson stainings (scale bar = 100 µm). Red arrow (growth epiphyseal line); Yellow arrow (bone trabeculae); Black arrow (fat cells); *Vs. Con group, *P* < 0.05, ^#^Vs. LPS group, *P* < 0.05.

After 12 weeks of NaB intervention, we used Micro-CT scans and pathological sections to detect changes in bone trabecular bone mass and trabecular microstructure in the distal femur. Consistent with the expected results, this study also found serious bone trabecular loss in the epiphysis of LPS-treated rats, which was obviously confirmed by Micro-CT 3D reconstruction results (Figure 1). Meanwhile, quantitative results further confirm this fact (Figure 1).

HE and Masson staining observed that a large number of bone trabeculae disappeared in the LPS group, and the blank area around the epiphyseal line was filled with adipocytes (Figure 1C). A large number of bone trabeculae remained in the epiphysis of the femoral shaft of rats after NaB, and although the imaging and histological observations were not as abundant as those in the Con group, they were significantly improved compared with those in the LPS group (Figure 1). At the same time, quantitative results further confirm this performance (Figure 1). Micro-CT evaluation showed that the NaB treated-rats had higher BMD, BV/TV, Tb. Th, Tb. N, and Lower Tb. Sp compared to the LPS group (*P* < 0.05, Fig 1B).

**Figure 2.**
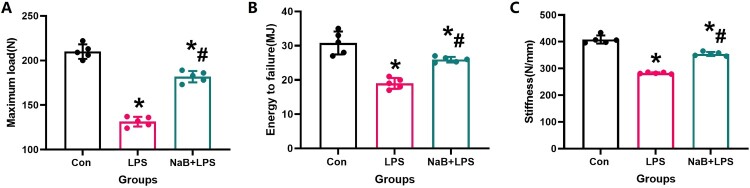
Systemic administration with NaB could increase the biomechanical parameters of the shaft of femur in LPS-treated rats expressed by biomechanical parameters such as maximum load (A), energy to failure (B), stiffness (C). *Vs. Con group, *P* < 0.05, #Vs. LPS group, *P* < 0.05.

In order to more intuitively understand the changes of bone quality in each treatment group, we conducted biomechanical experiments to observe the changes of femoral bone strength. By the three-point bending test, the bone strength was significantly impaired in the LPS group, showing a large reduction in energy-to-failure, maximum load, and stiffness parameters (*P* < 0.05, Figure 2), which is also responsible for the susceptibility of LPS patients to fracture. After 12 weeks of NaB treatment, the above parameters showed great improvement in LPS rats (*P* < 0.05, Figure 2). Based on the results observed at the biomechanical, histological and imaging levels, we can conclude that NaB treatment can improve the bone quality of LPS-treated rats under experimental conditions.

### 3.2. Nab recover bone metabolism in LPS-treated rats

**Figure 3.**
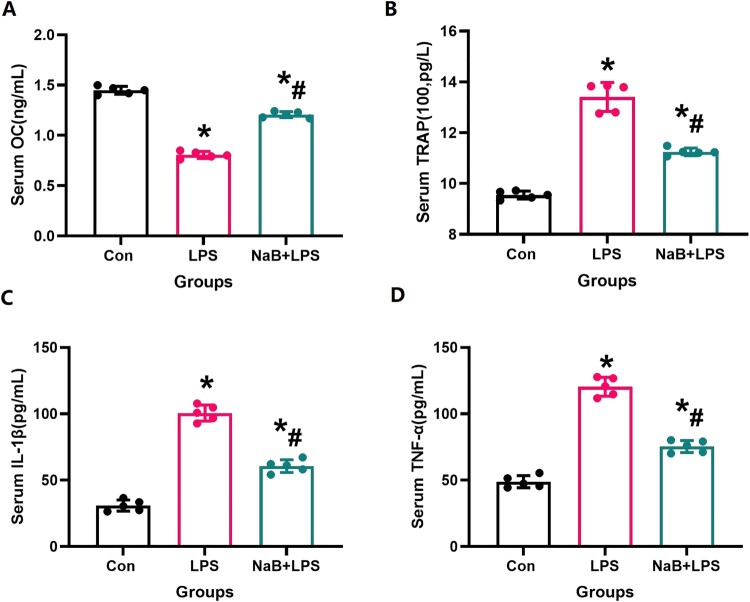
NaB therapy restores the broken balance of bone resorption and bone formation, and inflammatory due to LPS treatment (N = 5). A: Serum OC level; B: Serum TRAP level; C: Serum IL-1β level; D: Serum TNF-α level; *Vs. Con group, *P* < 0.05, #Vs. LPS group, *P* < 0.05.

To verify the influence of NaB on osteoblast and osteoclast, and inflammation in LPS-treated rats, we measured and compared the levels of serum IL-1β, TNF-α, TRAP and OC levels in all treatment groups. When compared with the LPS group, our results showed that NaB treatment significantly increased osteoblast activity and reduced osteoclast activity and inflammation (Figure 3, *P* < 0.05).

### 3.3. Nab reduces oxidative stress and inflammation in LPS-treated rats

**Figure 4.**
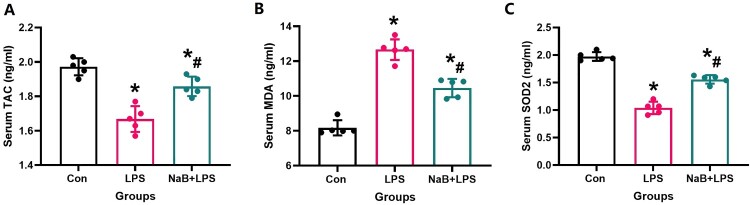
NaB therapy helps restore elevated levels of oxidative stress caused by LPS treatment (N = 5). A: Serum TAC level; B: Serum MDA level; C: Serum SOD2 level. *Vs. Con group, *P* < 0.05, #Vs. LPS group, *P* < 0.05.

**Figure 5.**
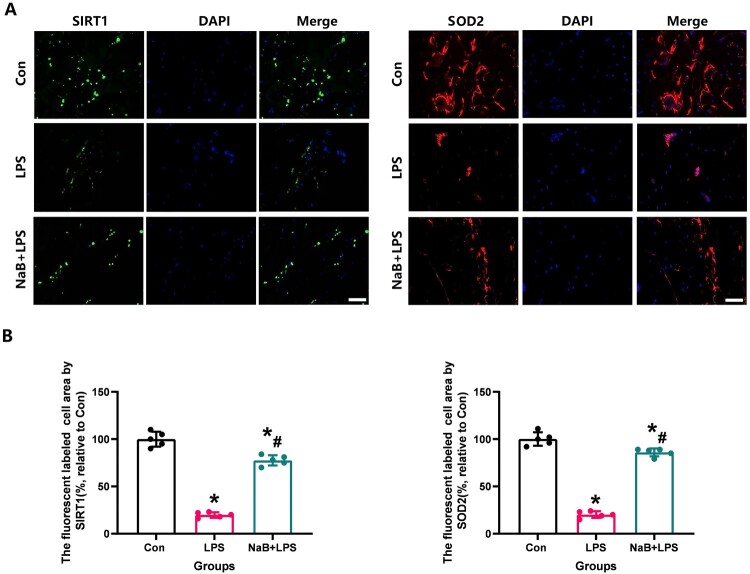
Systemic administration with NaB could alter the intensity of SIRT1 and SOD2 assessed by immunofluorescence in bone tissue in LPS treatment rats. A: Representative SIRT1 and SOD2 immunofluorescence staining of femoral bone in each group (Scale bar = 25 µm). B: Results of quantitative analysis of SIRT1 and SOD2 fluorescently marked bone tissue in different groups. N = 5 specimens/group. *Vs. Con group, *P* < 0.05, #Vs. LPS group, *P* < 0.05.

**Figure 6.**
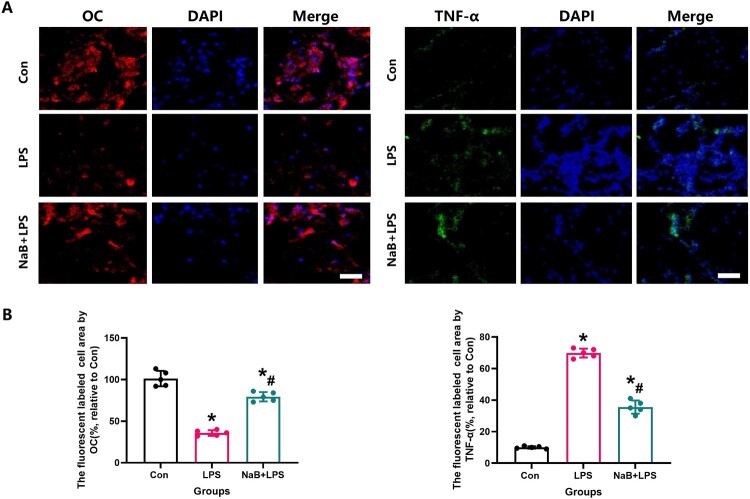
Systemic administration with NaB could alter the intensity of OC and TNF-α assessed by immunofluorescence in bone tissue in LPS treatment rats. A: Representative OC and TNF-α immunofluorescence staining of femoral bone in each group(Scale bar = 25 µm). B: Results of quantitative analysis of OC and TNF-α fluorescently marked bone tissue in different groups. N = 5 specimens/group. *Vs. Con group, *P* < 0.05, #Vs. LPS group, *P* < 0.05.

Since oxidative stress and inflammation play a crucial role in osteoporosis, we also detected changes in serum specific indexes of oxidative stress in rats from all treatment groups. When compared with the LPS group, our results showed that NaB treatment significantly increased TAG and SOD2 levels, and reduced MDA activity (Figure 4, *P* < 0.05). Previous studies have confirmed that the SIRT1/SOD2 signaling pathway is closely related to oxidative stress [31,32,29]. To verify the role of NaB on oxidative stress and inflammation in LPS rats, the expression of SIRT1, TNF-α and SOD2 was used by immunofluorescence staining to indirectly confirm the level of oxidative stress and inflammation (Figures 5 and 6). Our results showed that compared with the LSP group, the immunofluorescence intensity of SIRT1 and SOD2 in the bone tissue of rats treated with NaB were significantly increased, while the immunofluorescence intensity of TNF-α was significantly decreased (*P* < 0.05). We also observed that NaB can promote the expression of OC in rats treated with LSP. These results suggest that NaB's promotion of bone formation may be related to the reduction of oxidative stress and inflammation.

### 3.4. Nab protects the osteogenic potential in LPS intervene environments.

**Figure 7.**
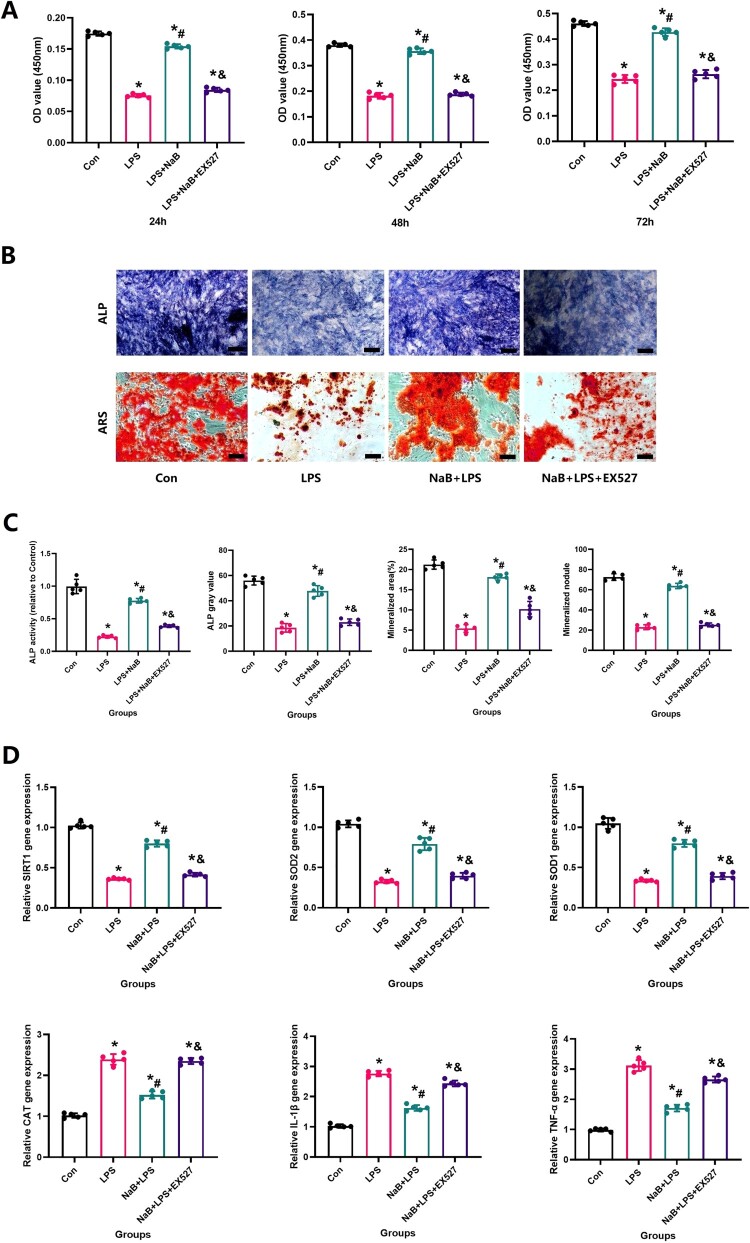
NaB therapy protects osteogenic activity in the presence of LPS treatment. A: ⁣NaB therapy can protect MC3T3-E1 cells from decreased activity due to LPS treatment via CCK-8 assay. B: ⁣ALP and RES stainings were observed at high magnification to assess the ability of NaB to significantly improve ALP expression and the formation of calcified nodules in LPS-treated MC3T3-E1 (Scale bar = 50 µm). C: ⁣The changes in ALP expression and the ability to form calcified nodules in LPS-treated MC3T3-E1 after NaB intervention were quantitatively assessed, including ALP gray value, ALP activity, mineralized areas and mineralized nodules (*N* = 5). D: Quantitative expression of target genes was detected by RT-PCR. *Vs. Con group, *P* < 0.05, #Vs. LPS group, *P* < 0.05, &Vs. NaB + LPS group.

**Figure 8.**
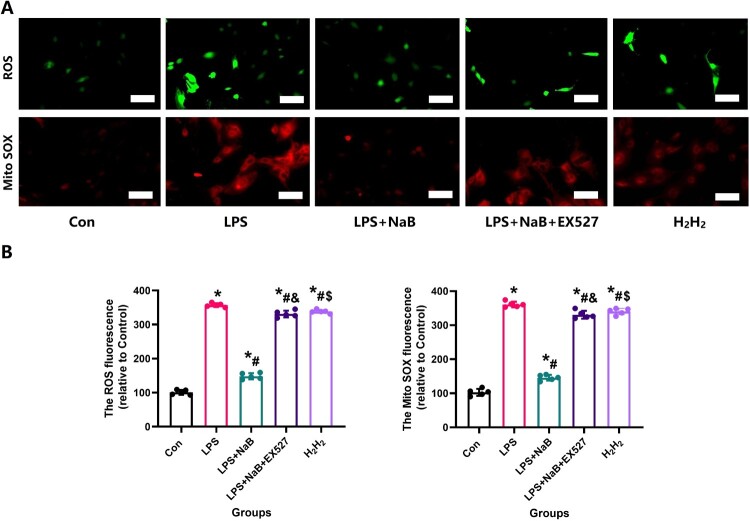
NaB therapy protects MC3T3-E1 from impaired osteogenic potential due to high oxidative stress induced by LPS. A: ⁣NaB therapy reduces the intracellular oxidative stress that causes damage in MC3T3-E1 due to LPS treatment as assessed by ROS and MitoSOX (Scale bar = 25 µm); B: ROS and MitoSOX fluorescence staining intensity of MC3T3-E1 cells in each group. *Vs. Con group, *P* < 0.05, #Vs. LPS group, *P* < 0.05, &Vs. NaB + LPS group, *P* < 0.05, $Vs. NaB + LPS + EX527 group.

**Figure 9.**
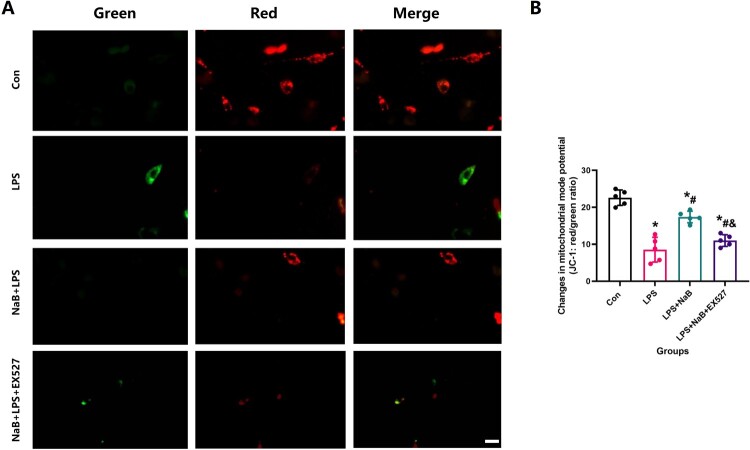
NaB therapy promotes the recovery of mitochondrial membrane potential activity in the presence of LPS by JC-1 staining. A: JC-1 stainings were observed at high magnification to assess the ability of NaB to significantly improve the recovery of mitochondrial membrane potential in LPS-treated MC3T3-E1 (Scale bar = 25 µm); B: ⁣Quantitative analysis of changes in mitochondrial membrane potential in LPS-treated MC3T3-E1 after NaB intervention. *Vs. Con group, *P* < 0.05, #Vs. LPS group, *P* < 0.05, &Vs. NaB + LPS group.

In order to more directly observe the effect of NaB on bone remodeling, we performed in vitro experiments to see the protective effect of NaB on osteogenic differentiation and function in the presence of LPS. CCK-8 experiment showed that LPS could significantly inhibit MC3T3-E1 proliferation, however, NaB treatment restored osteoblast proliferation (Figure 7A). The reduction of fluorescence intensity of Mito SOX and ROS also confirmed that NaB treatment can significantly reduce the level of cellular oxidative stress (Figure 7 B and C). RT–PCR results showed that the expression of SOD1, SOD2 and SIRT1 genes were significantly increased in NaB treatment group, while CAT, IL-1β and TNF-α expression were significantly increased (Figure 7D). In addition, AR and ALP staining indicated that the ALP activity was significantly increased after NaB treatment, and the ability to form calcified nodules was also significantly increased (Figure 8A and B). Besides, JC-1 staining suggests that hyperglycemia may lead to an abnormal mitochondrial membrane potential, while NaB may promote the restoration of a normal mitochondrial membrane potential (Figure 9).

**Figure 10.**
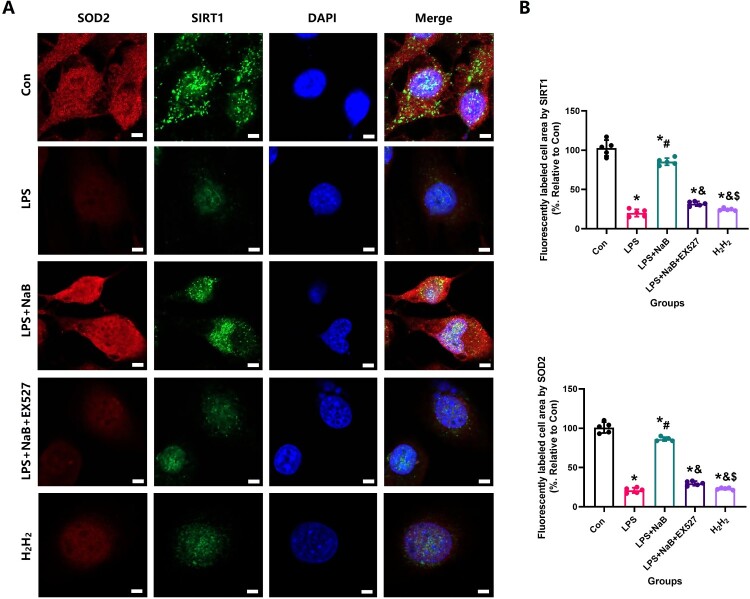
NaB therapy protected MC3T3-E1 from impaired resistance to oxidative stress due to LPS treatment and was analyzed by SIRT1 and SOD2 expression. A: SIRT1 and SOD2 were observed at high magnification to assess the ability of NaB to significantly improve the resist oxidative stress in LPS-treated MC3T3-E1 (Scale bar = 10 µm); B: ⁣Quantitative analysis of changes in SIRT1 and SOD2 expression in LPS-treated MC3TE-E1 after NaB intervention. *Vs. Con group, *P* < 0.05, #Vs. LPS group, *P* < 0.05, &Vs. NaB + LPS group, *P* < 0.05, $Vs. NaB + LPS + EX527 group.

Since SIRT1 and SOD2 play a critical role in regulating osteoblastic activity and the ability to resist oxidative stress, we furthermore performed immunofluorescence staining of SIRT1 and SOD2 on the treated cells. LPS may lead to a decreased fluorescence intensity of SIRT1 and SOD2, while the increase of SIRT1 and SOD2 fluorescence intensity visually showed that NaB treatment could significantly increase the cellular antioxidant stress ability (Figure 10). However, EX527 removed the positive effect of NaB on MC3T3-E1 in the LPS presence environment. These results suggest that NaB promotes osteoblast proliferation, differentiation and function by inhibiting osteoblast oxidative stress via SIRT1 signal activation.

### 3.5. NaB inhibits osteoclast differentiation in RAW264.7 cells treated with LPS

**Figure 11.**
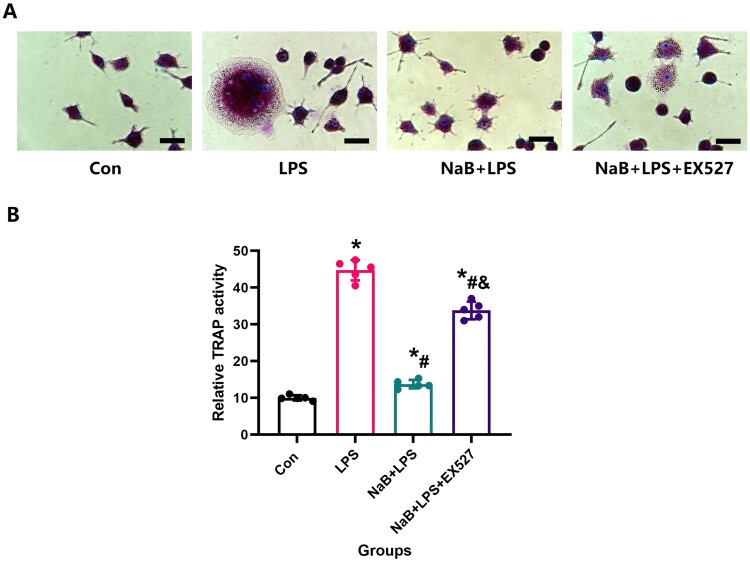
NaB treatment inhibited osteoclast differentiation in RAW264.7 cells treated with LPS. A: Representative TRAP staining showed the ability of RAW264.7 cells in each group to differentiate into osteoclasts (Scale bar = 25 µm); B: ⁣Quantitative analysis of changes in TRAP expression after NaB intervention. *Vs. Con group, *P* < 0.05, #Vs. LPS group, *P* < 0.05, &Vs. NaB + LPS group.

To more directly observe the effect of NaB on bone resorption, we conducted in vitro experiments to observe the effect of NaB on the osteoclast differentiation of RAW264.7 cells treated with LPS. TRAP staining indicated that the osteoclastic differentiation activity was significantly increased after LPS treatment, and the ability to differentiate into osteoclasts in NaB + LPS was also significantly decreased (Figure 11).

## 4. Discussion

Currently, a gradually increasing number of studies have now reported the beneficial effects of NaB on the prevention of bone loss. However, there have been fewer reports of protective effects of NaB on bone loss induced by LPS treatment. Therefore, the aim of this study is to explore whether NaB treatment can reduce bone loss in LPS-treated rats. In the current study, we used standard LPS-treated animals to copy chronic inflammation observed in osteoporosis patients, and then examined the effect of NaB therapy on bone mass in LPS-treated rats. As we expected, the decreased levels of bone mass in LPS-treated animals has been observed in previous studies [33,34] consistent with results of our current study. Moreover, LPS therapy may also influence bone remodeling and formation and impair fracture healing [33,34]. Based on the above results, we can confirm that chronic inflammation is closely related to low bone mass. Therefore, it is necessary to promote bone formation to reduce potential bone loss in chronic inflammation state. The present findings support the hypothesis that the application of NaB results in a higher trabecular mass in the femoral metaphysis of LPS-treated rats.

Existed studies have shown that the imbalance in skeletal remodeling can be reversed by NaB through change bone metabolism and decreased bone turnover to prevent bone loss and improve bone formation in C57BL/6J mice [35]. Indeed, other studies have reported that application of NaB could protect local tissue from damage in some organs during hepatic diseases [36]. In the study, treatment with NaB facilitated new bone formation, as evidenced by increased microscopic parameters including BMD, BV/TV, Tb.Th, Tb.N, Conn.D in LPS-treated rats. To explore the role of NaB in bone remodeling and bone metabolism, we conducted a bone biomechanical analysis of the femur using a three-point bending test. Our data indicate that NaB could cause beneficial effects on bone mass and bone strength in LPS-treated rats.

⁣It is generally known that LPS could promote inflammation production, to a significant extent, is a major pathogenic factor in LPS-related organ injury and cell damage [37]. Simultaneously, it has been reported that ROS production could promote bone loss, while preventing ROS production could compensate the effects of postmenopausal osteoporosis [38]. Also, TNF-α and IL-1β are the most classical markers of pro-inflammatory cytokines and can be used directly to reflect the level of inflammation in LPS-induced inflammation [39]. The serum content of MDA along with TAC and SOD2 activities are widely used indicators reflecting the extent of cells and tissue damage [40,41]. Therefore, in this study, we also assessed the changes in TNF-α, IL-1β, SOD2, MDA and TAC in the treated group after NaB treatment and compared the results with those of LPS-treated rats in different treatment groups. The data showed a significant increase in serum TNF-α, IL-1β, MDA levels and a decrease in TAC and SOD2 levels in LPS-treated rats compared with control rats. In particular, NaB treatment prevented depletion of serum TAC and SOD2 levels and inhibited TNF-α, IL-1β, MDA elevation. Increased oxidative stress is expected to affect bone metabolism, as well as bone mass [40,41]. Similarly, we also observed a decrease in TRAP levels and an increase in serum OC levels in LPS-treated rats treated with NaB compared to LPS-treated rats.

⁣Since both osteoblasts and osteoclasts play a key role in maintaining bone mass and bone formation, in vitro studies were performed to assess the potential bone metabolism of NaB in RAW264.7 cells and MC3T3-E1 cells [42]. After the introduction of NaB, ALP activity and mineralization were significantly facilitated in MC3T3-E1 cells treated with NaB, as we hypothesized, indicating a positive effect of NaB on osteogenic differentiation in MC3T3-E1 cells in LPS treatment. Meanwhile, we also observed that LPS promoted the differentiation of RAW264.7 cells into osteoclasts as assessed by TRAP staining, while NaB inhibited LPS-osteoclast differentiation. Sirtuin1 (SIRT1), a considered anti-aging factor, is an evolutionarily conserved NAD + -dependent deacetylase, regulate a variety of cellular metabolic and aging processes [43]. ⁣Activation of the SIRT1 signaling pathway and up-regulated SOD2 expression protect mitochondria against oxidative stress in some bone metabolic diseases [44,45]. Therefore, it is believed that the SIRT1 and SOD2 might act as specific factors in oxidative stress, and the study the levels of SIRT1 and SOD2 is important for understanding the diseases caused by oxidative stress. In this study, the expression of SIRT1 and SOD2 proteins in osteoblasts was significantly reduced were observed in the LPS group. After NaB treatment, we observed a significant increase in the expression of SIRT1 and SOD2 proteins in NaB + LPS, which is conducive to improving the antioxidant capacity of cells. In light of the above positive results, we believe that LPS may repress osteoblastic differentiation and promote osteoclast differentiation by upregulating levels of oxidative stress and inflammation. Moreover, application of NaB induced osteoblast proliferation and differentiation, enhanced osteoblast growth, matrix mineralization, and inhibited osteoclast differentiation and activity, and thus improved bone quality in LPS rats.

## 5. Conclusions

Taken together, the results from our experiments confirmed that NaB can stimulate the ability of osteogenic proliferation and differentiation and inhibit osteoclast differentiation by inhibition of oxidative stress and inflammation, leading to a high-turnover bone loss and bone regeneration inhibition in LPS rats. However, this study did not further examine the changes in circulating cortisol and DHEA levels or explore whether the protective effect of bone mass was achieved by regulating histone deacetylase (HDAC) enzymes. Therefore, subsequent studies have addressed these shortcomings.

## Data Availability

The data that support the findings of this study are available from the corresponding author, [ZS Tao], upon reasonable request.
